# An Artificial Neural Networks Model for Early Predicting In-Hospital Mortality in Acute Pancreatitis in MIMIC-III

**DOI:** 10.1155/2021/6638919

**Published:** 2021-01-28

**Authors:** Ning Ding, Cuirong Guo, Changluo Li, Yang Zhou, Xiangping Chai

**Affiliations:** ^1^Department of Emergency Medicine, The Second Xiangya Hospital, Emergency Medicine and Difficult Diseases Institute, Central South University, China; ^2^Department of Emergency Medicine, Changsha Central Hospital, University of South China, China

## Abstract

**Background:**

Early and accurate evaluation of severity and prognosis in acute pancreatitis (AP), especially at the time of admission is very significant. This study was aimed to develop an artificial neural networks (ANN) model for early prediction of in-hospital mortality in AP.

**Methods:**

Patients with AP were identified from the Medical Information Mart for Intensive Care-III (MIMIC-III) database. Clinical and laboratory data were utilized to perform a predictive model by back propagation ANN approach.

**Results:**

A total of 337 patients with AP were analyzed in the study, and the in-hospital mortality rate was 11.2%. A total of 12 variables that differed between patients in survivor group and nonsurvivor group were applied to construct ANN model. Three independent variables were identified as risk factors associated with in-hospital mortality by multivariate logistic regression analysis. The predictive performance based on the area under the receiver operating characteristic curve (AUC) was 0.769 for ANN model, 0.607 for logistic regression, 0.652 for Ranson score, and 0.401 for SOFA score.

**Conclusion:**

An ANN predictive model for in-hospital mortality in patients with AP in MIMIC-III database was first performed. The patients with high risk of fatal outcome can be screened out easily in the early stage of AP by our model.

## 1. Background

Current evidence showed that around 20-30% patients with acute pancreatitis (AP) developed into severe acute pancreatitis (SAP) with high mortality [1]. An early accurate evaluation of severity and prognosis of AP, especially at the time of admission is significant for physicians to take many attentions and more effective managements to the patients whose physical condition may be likely to getting worse.

Previous studies illuminated that some laboratory variables such as red cell distribution width (RDW) [2] and hematocrit (HCT) [3] and several scoring systems including Ranson [4] and sequential organ failure assessment (SOFA) [5] were applied to evaluate the prognosis of AP. Due to the fluctuation in accuracy of single laboratory variable, the predictive performance could be affected. Moreover, both Ranson and SOFA scores include around ten variables and need to be recorded dynamically; the availability of Ranson and SOFA scores in early prediction has been limited. Hence, it is necessary to construct an early predictive model with better accuracy.

Artificial neural networks (ANN), which were on the basis of function of biological neural networks, have been successfully applied in clinical assessment and decision-making in different disorders such as early detection of bacteremia [6], outcomes of pelvic organ prolapse [7], and predicting prostate cancer on initial biopsy [8]. In this study, we aimed to systematically assess the predictive performance of ANN in association of different variables on admission with in-hospital mortality in patients with AP in a publicly accessible database of Medical Information Mart for Intensive Cart III (MIMIC-III).

## 2. Methods

### 2.1. Dataset

In this study, patients diagnosed with acute pancreatitis (AP) in MIMIC-III were enrolled. MIMIC-III database was a large US-based critical care database, which contained data associated with 53,423 adult patients (aged 16 years or above) from 2001 to 2012 and 7870 neonates from 2001 to 2008 in intensive care unit (ICU) [9]. Data including vital signs, medications, laboratory measurements, observations and notes charted by care providers, fluid balance, procedure codes, diagnostic codes, imaging reports, hospital length of stay, and survival data were comprehensively recorded. The following tables from MIMIC-III dataset were utilized in our study: ADMISSIONS, CHARTEVENTS, D_ICD DIAGNOSIS, D_ITEMS, D_LABIEVENTS, DIAGNOSIS_ICD, ICUSTAYS, LABEVENTS, NOTEEVENTS, PATIENTS, INPUTEVENTS_CV, INPUTEVENTS_MV, and OUTPUTEVENTS.

### 2.2. Definition

When at least two of the following criteria were confirmed, the AP was diagnosed. First, abdominal pain associated with AP; second, the levels of amylase and/or lipase increased at least 3-times above the normal threshold; third, ultrasonography and/or CT scanning showed significant image of AP. Only the data of each patient in the first admission were utilized in this study. Patients with missing >5% individual data and age less than 18 were excluded.

### 2.3. Data Extraction

Structure query language was used for data extraction from MIMIC-III database. General information including age, sex, marital status, and ethnicity were collected. Clinical and laboratory variables were collected within 24 hours after admission including systolic blood pressure, diastolic blood pressure, heart rate, white blood cells (WBC), platelet (PLT), mean corpuscular volume (MCV), hematocrit (HCT), glucose, prothrombin time (PT), thrombin time (TT), albumin, creatine kinase MB isoenzyme (CK-MB), alanine aminotransferase (ALT), aspartate aminotransferase (AST), total bilirubin, creatinine, amylase, lipase, lactate dehydrogenase (LDH), total calcium, sodium, anion gap, lactate, and triglyceride. When one variable was recorded in different time of initial 24 hours, the first one was enrolled in our study. The scores of SOFA and Ranson were calculated to assess the severity of AP patients on the basis of the data in MIMIC-III, respectively. Clinical outcomes were length of stay (LOS) in ICU and in-hospital mortality.

### 2.4. ANN Model Development

The back propagation (BP) ANN model was performed for predicting in-hospital mortality in patients with AP, which was composed of three layers of nodes arranged in series: an input layer, a hidden layer, and an output layer [10]. The variables, which were identified to be significant difference by univariate analysis, were included in the input layer and applied to develop the model. The hidden layer contained several unobservable nodes or units, which were associated with functions of the predictors in partly depending on the network type and user-controllable specifications. The output layer contained the predicted pattern or outcomes (in-hospital mortality). The feed-forward form established that the connections from the input layer to the output layer without any feedback loop. The error back propagation was utilized as a learning rule to adjust the model [11]. The entire group was divided into training group (80%) and validation group (20%). The ANN performed its predicted model based on the input variables, and, then, the synaptic weights were adjusted to minimize the discrepancy between the actual and the predicted model, which was realized by calculating the error for every neuron in the network. Each synaptic weight was identified by two factors including the activity of the neuron projecting from and the error of the neuron projecting to [12].

### 2.5. Statistical Analysis

Continuous data were expressed as median with interquartile range (IQR) or range and were compared using the Wilcoxon rank-sum test or the Wilcoxon signed-rank test for paired data. Categorical data were compared using the Fisher test or Chi-squared tests. Univariate and multivariate logistic regression was applied to perform logistic regression model. Receiver-operating characteristic (ROC) curve was analyzed for comprehensively evaluating the predicting capability of the models. The predictive capability of models was also assessed by positive predictive value (PPV), negative predictive value (NPV), sensitivity, and specificity ranging from 0 to 1, which represented from the lowest performance to the highest performance.

Statistical analysis was performed using the SPSS software (version 26), and ANN model was performed with PyTorch (version1.2.0). A *P* value of less than 0.05 was defined as statistical significance.

## 3. Results

### 3.1. Patients

**Figure 1:**
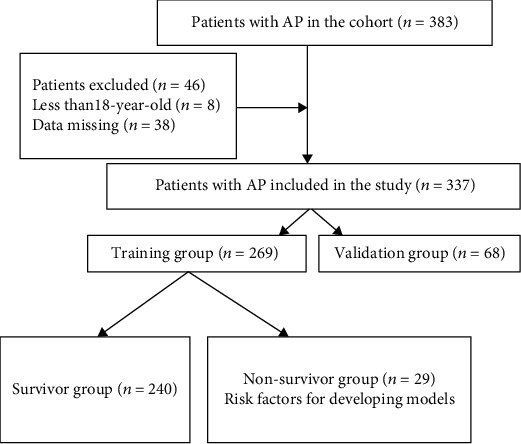
Flow chart for patients' enrollment and study design. Abbreviations: AP: acute pancreatitis.

Initially, a total of 383 patients with AP were identified and 46 patients including 8 patients with age less than 18 and 38 patients with data missing were excluded (Figure 1). Finally, 337 patients with general characteristics were included in this study (Table 1). The median age of the patients was 65, while the proportion of males was 56.08%. Nearly half were married and white patients accounted for 65.88%. The median day of LOS in ICU was 8.6, and the in-hospital mortality rate was 11.2%. The median scores of SOFA and Ranson were 1 and 3, respectively.

### 3.2. Comparison Baseline Characteristics between Survivor and Nonsurvivor Groups

Baseline characteristics for survivor and nonsurvivor groups were demonstrated in Table 2. In general, there was no significant difference in the proportion of males, systolic blood pressure, diastolic blood pressure, and heart rate between the two groups, while patients in nonsurvivor group were older (*P* = 0.035).In nonsurvivor group, the levels of WBC (*P* < 0.001), HCT (*P* = 0.048), PT (*P* < 0.001), CK-MB (*P* = 0.025), ALT (*P* = 0.037), total bilirubin (*P* < 0.001), creatinine (*P* = 0.003), amylase (*P* = 0.046), lipase (*P* = 0.033), and lactate (*P* = 0.035) were significantly higher than those in survivor group, while the level of total calcium (*P* = 0.041) in nonsurvivor group was lower. There were no significant differences in other laboratory variables such as PLT, MCV, glucose, TT, albumin, AST, LDH, sodium, anion gap, and triglyceride between the two groups. Patients in nonsurvivor group had longer LOS in ICU (*P* = 0.002) and higher scores of SOFA and Ranson (both *P* < 0.001).

### 3.3. Multivariate Logistic Regression Analysis of Variables Associated with In-Hospital Mortality

Three independent variables were identified as risk factors associated with in-hospital mortality by multivariate logistic regression analysis (Table 3): ALT (odds ratio (OR) = 1.005, 95% CI: 1.000-1.009), WBC (OR = 1.296, 95% CI: 1.074-1.565), and total calcium (OR = 0.336, 95% CI: 0.175-0.645).

### 3.4. ANN Model Development

**Figure 2:**
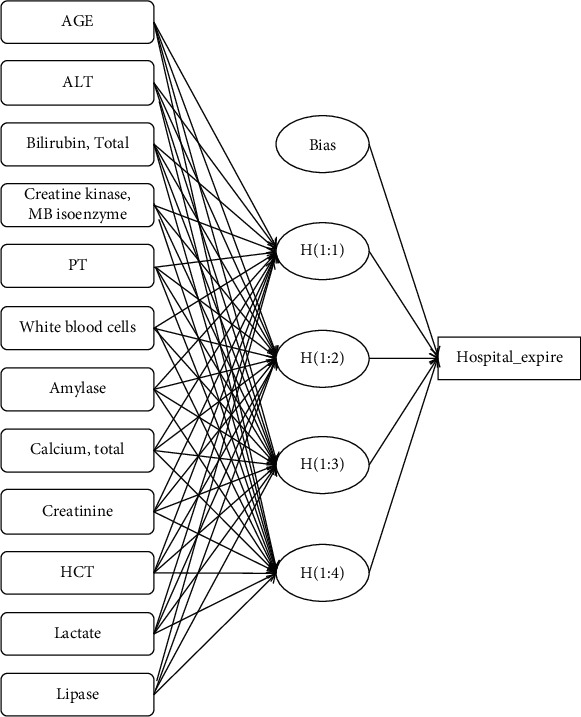
The structure of artificial neural networks. Abbreviations: ALT: alanine aminotransferase; PT: prothrombin time; HCT: hematocrit.

**Figure 3:**
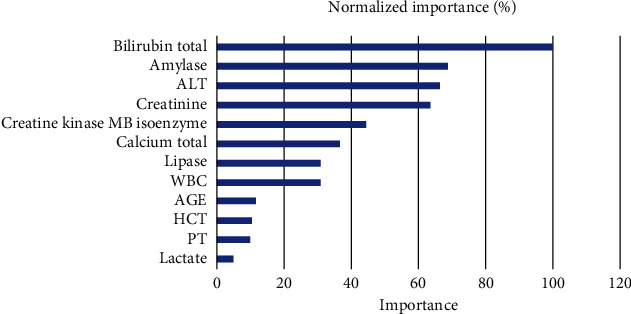
The normalized importance of 12 variables for predicting in-hospital mortality by artificial neural networks. Abbreviations: ALT: alanine aminotransferase; PT: prothrombin time; HCT: hematocrit; WBC: white blood cell.

The training group was utilized to develop ANN model. Baseline characteristics of the training group and validation group were demonstrated in Table 4. It showed that the two groups were well balanced in the distribution of clinical characteristics. 12 variables selected by univariate analysis including age, ALT, total bilirubin, CK-MB, PT, WBC, amylase, total calcium, creatinine, HCT, lactate, and lipase were consisted the input layer. The output layer was the prediction of in-hospital mortality (hospital expire) (Figure 2). In the ANN model, total bilirubin, amylase, ALT, and creatine were the top four of important variables for predicting in-hospital mortality, with a normalized importance of 100%, 68.8%, 66.0%, and 63.3%, respectively (Figure 3). When the model was applied to validation group, it had a sensitivity of 0.666, specificity of 0.661, PPV of 0.563, and NPV of 0.916 (Table 5).

### 3.5. Predictive Performance of Different Models

**Figure 4:**
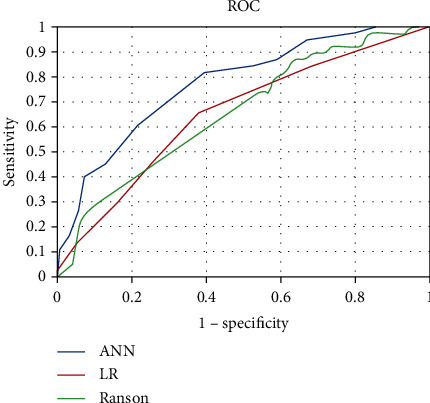
The receiver operating characteristic curves of ANN, LR, and Ranson in predicting in-hospital mortality. Abbreviations: ANN: artificial neural network; LR: logistic regression.

The evaluating indexes including accuracy, PPV, NPV, sensitivity, specificity, and the area under the ROC curve (AUC) of ANN, logistic regression (LR), Ranson, and SOFA for the prediction of in-hospital mortality in AP were illuminated in Table 5. The accuracy of predictive performance in ANN, LR, Ranson score, and SOFA score was 0.662, 0.660, 0.626, and 0.415, respectively. The predictive performance based on the AUC was 0.769 for ANN model, 0.607 for LR, 0.652 for Ranson score, and 0.401 for SOFA score, respectively. AUCs only >0.5 included in Figure 4 demonstrated that the overall performance of ANN was the best.

## 4. Discussion

Acute pancreatitis, as a common digestive disorder, varied in clinical course based on different clinical characteristics of different individuals [1], some of which could be totally recovery shortly, while others' condition may be deteriorating from a mild disease to a life-threatening illness with poor outcomes. Early identification of patients with AP who are likely to get high risk of worse prognosis is crucial for early intervention and management so that special medical therapy can be implemented as early as possible, which could significantly improve clinical outcomes.

So far, there have been several scoring systems for evaluating the severity and prognosis of AP [13], while few of scoring systems have been utilized for predicting in-hospital mortality in AP. It has been reported that Ranson score, as a predictive score for several decades in AP, had a good performance in predicting in-hospital mortality in SAP when score ≥4 with an AUC of 0.94 [14]. Another study showed that Ranson score ≥2.5 was a predictor for 28-day mortality in SAP [2]. SOFA score was mainly applied to assess organ disfunction [15]. In a retrospective research on AP, dynamically assessing SOFA score was better for clinical decision-making. While in one week after admission, the AUC of SOFA in predicting all-cause mortality and in-ICU mortality were 0.858 and 0.944, respectively [16].

In our research, ANN model for predicting in-hospital mortality in AP was performed, and the comparison between different models was analyzed. For early prediction, ANN with an AUC of 0.769 was superior compared to LR, Ranson score, and SOFA score. Ranson score had an AUC of less than 0.7, and its score needed at least 48 hours after admission to be calculated so that it was unable to predict in-hospital mortality at the early stage of AP, especially within 24 hours after admission. SOFA score showed a relatively low AUC of less than 0.5 in this study, which could not be early predicting in-hospital mortality in this study. Researches proved that organ dysfunction in AP was usually easily detected and resolved at early clinical stage so that it did not have a significant impact on the mortality, while worsening of organ dysfunction was related to the poor prognosis and mortality [17]. That could partly explain why SOFA score did not show a good performance in predicting in-hospital mortality in our study. Logistic regression, as a statistical modeling method for constructing the association of the probability of clinical outcome with various potential predictors, has been widely utilized [18]. In our study, logistic regression model with three independent variables including ALT, WBC, and total calcium was constructed. However, the predictive performance was not very satisfactory. It could be explained by that there were asymmetry of nonnormal data and relatively small samples especially the low incidence of nonsurvivors in this cohort.

ANN is a computer model which mimics the human brain with parallel, nonlinear computational elements arranged in several layers [19]. Compared with traditional statistical models such as logistic regression, ANN model can enroll time-dependent factors and nonlinear factors which are related to the prognosis, automatically deal with missing values, and realize feature selection. Recently, ANN model has been applied in some researches in AP even in a few of clinical studies with small samples. In a retrospective study with 263 patients with SAP and moderately SAP, ANN predicted intra-abdominal infection precisely with a ROC of above 0.8 [20]. In the prediction of acute lung injury following SAP, ANN model was also a valuable tool [21]. A research on predictive clinical outcomes in a cohort with only 92 AP patients also showed that ANN was superior to the Ranson score and Balthazar grading systems in CT [22]. In this study, twelve variables including age, ALT, total bilirubin, CK-Mb, PT, WBC, amylase, total calcium, creatinine, HCT, lactate, and lipase which were identified to be significant difference by univariate analysis were set as the input layers, and the most important four variables including total bilirubin, amylase, ALT and creatine were identified, which also were reported in other studies. Evidence showed that the levels of total bilirubin and ALT were significantly higher in AP patients with a Ranson score ≥3 [23], and early elevated creatinine within 24 hours after admission was a good predictor of fatal outcomes in AP patients [24]. Serum level of amylase was not only for AP diagnosis but also associated with severity and prognosis of AP [25, 26].

The strength of this study was that an ANN predictive model for in-hospital mortality in patients with AP in MIMIC-III database was first performed and showed good performance even in relatively small samples when analyzing nonlinear interactions among different variables. MIMIC-III database was a large US-based critical care database. Hence, the severity of AP could be worse which may lead to a higher in-hospital mortality. So, our ANN model could be a guide for clinical management in AP, especially in SAP. When a patient with AP who was likely to develop severe condition with high risk for fatal outcome was detected on the basis of our ANN model, a series of managements would be implemented early in order to prevent organ disfunction and reduce the occurrence of complications such as lung infection and pancreatic necrosis.

Several limitations also should be clarified. First, ANN model was performed with relatively small samples in MIMIC-III. So further research with larger samples in multiple-center should be explored for validating and testing our model in order to enhance the application of the ANN model for predicting complications and prognosis of AP. Second, most of the patients in MIMIC-III were American, so cautions must be considered while applying the model to other ethnic patients. MIMIC-III database only included patients from 2001 to 2012. From 2012, the guidelines of acute pancreatitis have been developed, so there would be some limitations when our model has been applied. Third, it was a retrospective public database study. It was inevitable for missing part of data, and not all the variables which were associated with poor prognosis were recorded in the database. Due to the limitation of MIMIC-III database, some other important factors including IL-6 could not be included. The ANN model was constructed by the variables which were extracted from the MIMIC-III database. Through the analysis of the existing data by our ANN model, the model still had a good sensitivity and specificity. However, other variables collected prospectively also should be considered for future ANN model development in order to comprehensively evaluate the association of different clinical and laboratory characteristics with clinical outcomes in AP as well as avoid possible bias in the factors of treatment and patient.

## 5. Conclusion

In conclusion, an ANN predictive model for in-hospital mortality in patients with AP in MIMIC-III database was first performed. The patients with high risk of fatal outcome can be screened out easily in the early stage of AP by our model.

## Figures and Tables

**Table 1:** General characteristics of AP patients in MIMIC-III.

Characteristics	
Number of patients	337
Age (IQR, year)	65 (47.2-74)
Sex	
Male (*n*, %)	189 (56.08%)
Female	148 (43.92%)
Marital status (*n*, %)	
Divorced	24 (7.12%)
Married	160 (47.48%)
Single	96 (28.49%)
Others	57 (16.91%)
Ethnicity (*n*, %)	
Asian	5 (1.48%)
Black/African American	32 (9.50%)
Hispanic or Latino	9 (2.67%)
White	222 (65.88%)
Others	69 (20.47%)
In-hospital mortality (*n*, %)	38 (11.2%)
Length of stay in ICU (IQR, day)	8.6 (2-10)
Scoring system (IQR)	
SOFA	1 (0-3)
Ranson	3 (2-4)

Abbreviations: AP: acute pancreatitis; SOFA: sequential organ failure assessment.

**Table 2:** Univariate analyses of variables associated with in-hospital mortality.

Baseline variables	Survivor (*n* = 299)	Nonsurvivor (*n* = 38)	*p* value
Age (IQR, years)	57.75 (46.33-70.63)	74.79 (61.52-82.23)	0.035
Male (*n*, %)	165 (55.18%)	24 (63.15%)	0.195
Systolic blood pressure (IQR, mm Hg)	133 (117.5-149.5)	122.5 (109.5-154.5)	0.322
Diastolic blood pressure (IQR, mm Hg)	76 (64.5-87)	67 (51.25-95.5)	0.236
Heart rate (IQR, beats/min)	101 (85-118)	97 (87.25-117.75)	0.726
WBC (IQR, ×109/L)	13.1 (9.1-17.6)	16.55 (12.63-22.4)	<0.001
PLT (IQR, ×109/L)	228 (176-308)	234 (195.75-331.25)	0.532
MCV (IQR, fL)	90 (87-95)	89 (85-94.75)	0.123
HCT (IQR)	0.378 (0.327-0.42)	0.351 (0.313-0.401)	0.048
Glucose (IQR, mg/dL)	137 (105.25-184.75)	156 (120-235)	0.656
PT (IQR, s)	13.6 (12.8-14.9)	14.7 (13.63-16)	<0.001
TT (IQR, s)	27.7 (24.6-31.3)	30.7 (27.15-33.58)	0.12
Albumin (IQR, g/dL)	3.2 (2.7-3.9)	2.9 (2.25-3.35)	0.052
Creatine kinase, MB isoenzyme (IQR, IU/L)	4 (2-5)	5 (2-8.25)	0.025
ALT (IQR, IU/L)	44 (22-128.5)	49 (23.25-185)	0.037
AST (IQR, IU/L)	57 (30-131)	74.5 (37.75-228.75)	0.301
Total bilirubin (IQR, mg/dL)	0.9 (0.5-1.8)	1.45 (0.63-3.9)	<0.001
Creatinine (IQR, mg/dL)	1 (0.7-1.4)	1.55 (0.93-2.8)	0.003
Amylase (IQR, IU/L)	285 (92-786)	324 (92.25-1088.5)	0.046
Lipase (IQR, IU/L)	557 (99-1754)	656 (88.25-1248.25)	0.033
LDH (IQR, IU/L)	350 (240-517)	395 (257-597.5)	0.41
Total calcium (IQR, mg/dL)	8.3 (7.7-8.9)	7.85 (7.18-8.5)	0.041
Sodium (IQR, mmol/L)	139 (136-141)	139 (137-142)	0.444
Anion gap (IQR, mmol/L)	15 (13-18.75)	17 (14-20)	0.482
Lactate (IQR, mmol/L)	1.5 (1.2-2.4)	1.8 (1.23-2.88)	0.035
Triglyceride (IQR, mg/dL)	139.5 (94-225)	125 (70.5-227.25)	0.364
Length of stay in ICU (IQR, days)	3 (1.65-8.15)	12.16 (4.44-18.04)	0.002
SOFA (IQR)	1 (0-2.5)	2.5 (1.25-4)	<0.001
Ranson (IQR)	1 (1-2)	2 (2-3)	<0.001

Abbreviations: IQR: interquartile range; WBC: white blood cell counts; PLT: platelet; MCV: mean corpuscular volume; HCT: hematocrit; PT: prothrombin time; TT: thrombin time; ALT: alanine aminotransferase; AST: aspartate aminotransferase; LDH: lactate dehydrogenase; SOFA: sequential organ failure assessment.

**Table 3:** Multivariate logistic regression analysis of variables associated with in-hospital mortality.

						95% CI	
Variables	B	S.E	Wald	*p* value	OR	Lower	Upper
ALT	0.005	0.002	4.205	0.04	1.005	1.000	1.009
WBC	0.26	0.096	7.304	0.007	1.296	1.074	1.565
Total calcium	-1.09	0.333	10.726	0.001	0.336	0.175	0.645
Lactate	0.644	0.366	3.095	0.079	1.904	0.929	3.903

Abbreviations: WBC: white blood cell counts; ALT: alanine aminotransferase.

**Table 4:** Baseline characteristics between training group and validation group.

Baseline variables	Training group (*n* = 269)	Validation group (*n* = 68)	*p* value
Age (IQR, years)	65 (47-74)	64 (46-75)	0.412
Male (*n*, %)	148 (55.01%)	41 (60.2%)	0.532
Systolic blood pressure (IQR, mm Hg)	133 (113-143)	132.5 (114-142.5)	0.456
Diastolic blood pressure (IQR, mm Hg)	76 (72-81)	75 (71-80.5)	0.258
Heart rate (IQR, beats/min)	100 (85-118)	99.5 (84-117.5)	0.338
WBC (IQR, ×109/L)	13.4 (9.4-18)	13.55 (9.4-17.9)	0.217
PLT (IQR, ×109/L)	229 (176.5-309.5)	226 (173.5-311.5)	0.113
MCV (IQR, fL)	90 (86-95)	88 (85-94.5)	0.216
HCT (IQR)	0.375 (0.324-0.42)	0.383 (0.335-0.43)	0.109
Glucose (IQR, mg/dL)	140 (108-184)	138 (112-182)	0.214
PT (IQR, s)	13.7 (12.9-15.1)	13.8 (12.6-15)	0.315
TT (IQR, s)	27.8 (24.8-34.5)	27.6 (24.5-33.8)	0.146
Albumin (IQR, g/dL)	3.2 (2.7-3.8)	3.3 (2.7-3.9)	0.476
Creatine kinase, MB isoenzyme (IQR, IU/L)	4 (4-4)	4 (3.5-4)	0.517
ALT (IQR, IU/L)	45 (22.5-134.5)	47 (24.5-139)	0.108
AST (IQR, IU/L)	61 (30-138)	63 (31.5-136)	0.313
Total bilirubin (IQR, mg/dL)	0.9 (0.5-2)	0.9 (0.5-2.1)	0.447
Creatinine (IQR, mg/dL)	1 (0.7-1.7)	0.9 (0.7-1.8)	0.283
Amylase (IQR, IU/L)	287 (103.5-756.5)	290 (111-733.5)	0.102
Lipase (IQR, IU/L)	562 (99.5-1697)	555 (101.5-1675)	0.118
LDH (IQR, IU/L)	350.5 (258-500)	352 (257-498.5)	0.334
Total calcium (IQR, mg/dL)	8.2 (7.6-8.9)	8.1 (7.6-8.9)	0.618
Sodium (IQR, mmol/L)	139 (136-141)	138 (136-140)	0.337
Anion gap (IQR, mmol/L)	16 (13.5-19)	15.5 (13.5-18.5)	0.223
Lactate (IQR, mmol/L)	1.6 (1.3-2.1)	1.7 (1.3-2.0)	0.165
Triglyceride (IQR, mg/dL)	138 (118-175.5)	135 (117-174.5)	0.427
In-hospital mortality	29 (10.78%)	9 (13.23%)	0.315

Abbreviations: IQR: interquartile range; WBC: white blood cell counts; PLT: platelet; MCV: mean corpuscular volume; HCT: hematocrit; PT: prothrombin time; TT: thrombin time; ALT: alanine aminotransferase; AST: aspartate aminotransferase; LDH: lactate dehydrogenase; SOFA: sequential organ failure assessment.

**Table 5:** Predictive performance of different models.

	Accuracy	PPV	NPV	Sensitivity	Specificity	AUC
ANN	0.662	0.563	0.916	0.666	0.661	0.769
Logistic regression	0.660	0.360	0.919	0.346	0.923	0.607
Ranson	0.626	0.181	0.934	0.657	0.622	0.652
SOFA	0.415	0.140	0.939	0.815	0.364	0.401

Abbreviations: ANN: artificial neural networks; SOFA: sequential organ failure assessment; PPV: positive predictive value; NPV: negative predictive value; AUC: area under the ROC curve.

## Data Availability

The datasets used and/or analyzed during the present study were availed by the corresponding author on reasonable request.
